# Sensitivity of Ocular Trauma Score (OTS) classification to missing and undocumented prognostic variables: A robustness analysis in open globe injury

**DOI:** 10.1038/s41433-026-04738-0

**Published:** 2026-07-15

**Authors:** Poorvaprabha Patil, Soumya S Nair, Shailaja S Bhat, Sudha Menon, Shefali Misquith, Sulatha V Bhandary, Neetha IR Kuzhuppilly, Yogish Subraya Kamath, Vijaya H Pai

**Affiliations:** 1 Department of Ophthalmology, Kasturba Medical College, Manipal Academy of Higher Education, Manipal, India; 2 Department of Ophthalmology, Sree Uthradam Thirunal Academy of Medical Sciences, Thiruvananthapuram, India; 3 Department of Ophthalmology, MGM Muthoot Hospitals, Kozhencherry, India

**Keywords:** Eye diseases, Prognosis

Open globe injury (OGI) is a leading cause of preventable visual loss. Accurate early prognostication guides counselling, surgical prioritisation, and medico-legal communication. The Ocular Trauma Score (OTS), a widely used prognostication tool, assigns weighted deductions for presenting visual acuity and five adverse features: globe rupture, endophthalmitis, perforating injury, retinal detachment (RD), and relative afferent pupillary defect (RAPD) [1].

Reliable OTS computation requires complete variable documentation. In emergency practice, RAPD assessment is frequently hindered by media opacity or hyphaema, and posterior segment visualisation may be precluded by corneal oedema or absent B-scan facilities; RAPD has consequently been excluded from adapted scoring systems when it cannot be reliably assessed [2]. Undocumented variables are routinely treated as absent — introducing optimism bias when adverse predictors are unrecorded rather than truly absent. The robustness of OTS classification under such missing-data conditions has not been examined.

We conducted a retrospective robustness analysis of 16 consecutive OGI cases undergoing primary repair (Ethics Committee Approval No.: IEC2 186/2026). Baseline OTS was calculated per the published algorithm [1], with undocumented variables treated as absent. Three perturbation scenarios were applied: (1) RAPD assumed present in all globe rupture cases, reflecting established associations between OGI and afferent pathway dysfunction [3]; (2) occult RD assumed in patients with the lowest OTS visual acuity tier, modelling undetected posterior pathology [3]; and (3) both assumptions combined (RAPD + RD). Reclassification rates, Clopper–Pearson 95% confidence intervals, absolute agreement, Cohen’s *κ*, and Gwet’s AC1 were computed. AC1 addressed the prevalence paradox, whereby extreme marginal distributions artificially deflate *κ* [4]. The cohort size reflects the stringent inclusion criterion of complete baseline documentation; instability effects of this magnitude are not dependent on sample size for their existence, only for precision of their estimated frequency.

At baseline, 11 of 16 patients (68.8%) were Category 2, four (25.0%) Category 3, and one (6.3%) Category 4; none were Category 1. Under Scenario 1, 11 of 16 (68.8%; 95% CI: 41.3–89.0%) were reclassified — 10 shifting to Category 1; absolute agreement 31.2%, *κ* = 0.204, AC1 = 0.285. Under Scenario 2, 12 of 16 (75.0%; 95% CI: 47.6–92.7%) were reclassified; absolute agreement 25.0%, *κ* = 0.103, AC1 = 0.210. Under combined perturbation, 13 of 16 (81.3%; 95% CI: 54.4–96.0%) were reclassified; absolute agreement 18.8%, *κ* = −0.005, AC1 = 0.145. All shifts were downward, with progressive concentration into Category 1 (Table 1, and Fig. 1).

**Fig. 1:**
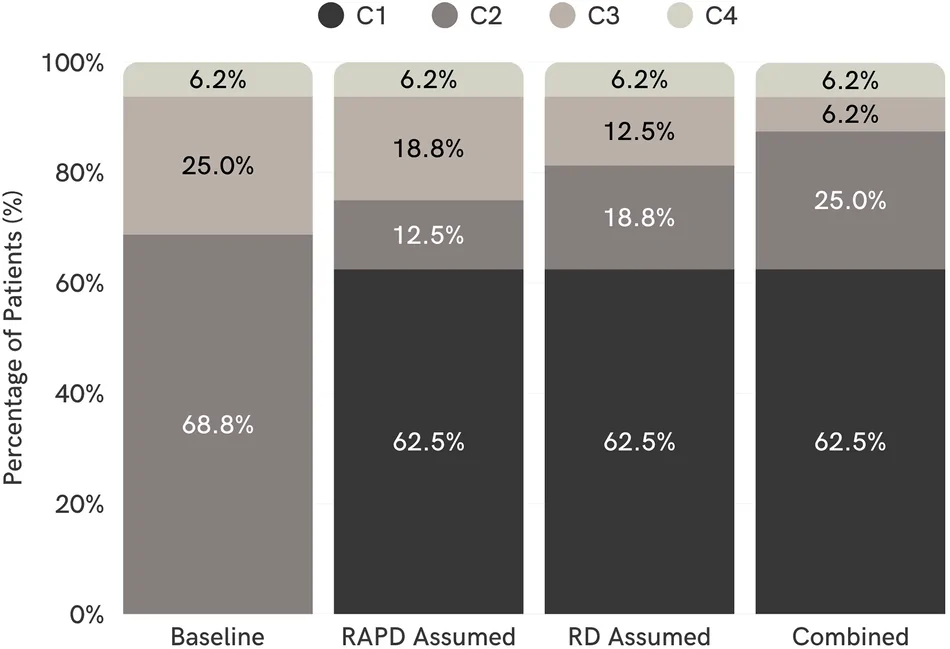
OTS category distribution at baseline and under three perturbation scenarios (*n* = 16). No patient was Category 1 at baseline; under all three scenarios, 62.5% shifted to Category 1 — additional reclassifications in Scenarios 2 and 3 reflect further downward movement within upper categories. All shifts were downward. (*RAPD*: relative afferent pupillary defect, *RD*: retinal detachment, *C1–C4*: OTS categories 1–4).

**Table 1:** Reclassification rates and agreement statistics under three predefined perturbation scenarios.

Scenario		*n* reclassified	Reclassification rate (95% CI)	Absolute agreement (%)	Cohen’s *κ*	Gwet’s AC1
1.	RAPD Assumed	11	68.8% (41.3–89.0%)	31.2%	0.204	0.285
2.	RD Assumed	12	75.0% (47.6–92.7%)	25.0%	0.103	0.210
3.	Combined (RAPD + RD)	13	81.3% (54.4–96.0%)	18.8%	−0.005	0.145

Agreement benchmarks: <0.00 poor, 0.00–0.20 slight, 0.21–0.40 fair, 0.41–0.60 moderate, 0.61–0.80 substantial, 0.81–1.00 almost perfect agreement.

*RAPD-* relative afferent pupillary defect, *RD-* retinal detachment, *VA-* visual acuity, *CI-* Clopper–Pearson confidence interval.

These findings demonstrate that OTS classification is highly sensitive to treating undocumented variables as absent. Under clinically plausible perturbations, over four-fifths of cases were reclassified and agreement fell to chance. The near-zero κ reflected distributional skew; Gwet’s AC1 confirmed genuine deterioration in reliability. These results do not challenge OTS’s predictive validity [1, 5] — they highlight that prognostic certainty depends on documentation completeness, not solely on the score’s discriminative properties.

Structured documentation of RAPD and posterior segment status — with explicit notation of examination uncertainty rather than silent omission — should be prioritised in trauma protocols. Future multicentre work should quantify undocumented variable prevalence and employ principled missing-data strategies such as multiple imputation [5] rather than defaulting to optimistic absent-variable assumptions.

## Data Availability

Anonymised OTS computation data and analysis scripts are available from the corresponding author on request.
